# Surgery after Neoadjuvant Chemotherapy: A Clip-Based Technique to Improve Surgical Outcomes, a Single-Center Experience

**DOI:** 10.3390/cancers14092229

**Published:** 2022-04-29

**Authors:** Carola Minella, Andrea Villasco, Marta D’Alonzo, Lisa Cellini, Francesca Accomasso, Silvia Actis, Nicoletta Biglia

**Affiliations:** 1 Academic Division of Obstetrics and Gynaecology, A.O. Ordine Mauriziano, University of Turin, 10128 Turin, Italy; carola.minella@unito.it (C.M.); andrea.villasco@unito.it (A.V.); marta.dalonzo@unito.it (M.D.); francesca.accomasso@unito.it (F.A.); silvia.actis@unito.it (S.A.); 2 Radiology Department, A.O. Ordine Mauriziano, 10128 Turin, Italy; lcellini@mauriziano.it

**Keywords:** breast cancer, neoadjuvant chemotherapy, conservative surgery, tissue markers, clip

## Abstract

**Simple Summary:**

Neoadjuvant chemotherapy (NACT) has an important role in the treatment of locally advanced breast cancer. After NACT, some lesions may be no longer visible at preoperative imaging, making breast and axillary conservative surgery more difficult. Among others, radiopaque clips are the most commonly used method to mark lymph nodes and tumor sites to tailor surgery in the post neoadjuvant setting.

**Abstract:**

Background: This study aims to describe the surgical management of breast cancer patients after neoadjuvant chemotherapy, with attention to the impact on surgical outcomes of a clip-based marking technique. Methods: Patients who underwent NACT at the Breast Unit of the A. O Ordine Mauriziano of Turin from January 2018 and had a surgical intervention by January 2022 were included. Data on the feasibility of clip insertion, after-treatment visibility, and successful removal during surgery were collected prospectively. Surgical outcomes in terms of breast-conserving surgery and axillary dissection reduction were described. Results: In 51 patients who had surgery after NACT, 55 clips were placed (34 breast and 21 axillary clips). Ultrasound visibility of the clips was optimal (91%) as well as preoperative localization and retrieval within the surgical specimen. Moreover, the use of the clip positively affected surgical outcomes. In our study, clip insertion allowed to avoid mastectomy and axillary dissection in patients with a complete radiological response. Conclusions: In our findings, the use of breast and/or lymph node clips has proved to be a simple and effective method to improve surgical conservative management of breast cancer patients after NACT.

## 1. Introduction

Breast cancer is the most frequent neoplasia in women, with more than 2 million new diagnoses worldwide each year [1]. Population screening based on periodical mammography has led to an increased rate of early diagnosis and a consequent reduction in breast cancer-specific mortality [2]. However, when patients are diagnosed with large tumors and nodal involvement, upfront surgery is not recommended. Neoadjuvant chemotherapy (NACT) has been shown to provide prognostic information and to allow a higher rate of conservative surgery, both on the breast and the axilla [3].

On the other hand, surgery after NACT can be complex, as the original lesions or involved lymph nodes can be no longer clinically detectable nor visible on preoperative imaging.

A radiological complete response (rCR) does not always correspond to a pathological complete response (pCR). For this reason, even if there are ongoing trials on the feasibility of surgery omission in selected patients after NACT [4], surgical intervention is still required.

### 1.1. Indications to Breast and Axillary Conservative Management

The possibility of breast-conserving surgery (BCS) after preoperative systemic treatment must be defined based on the initial presentation of the tumor and response to chemotherapy. In some cases (i.e., inflammatory breast cancer) BCS is contraindicated regardless of the response to NACT.

In the case of a radiological complete response, only the presence of a tissue marker guarantees the precise localization of the original site of the tumor increasing the BCS rate (up to 65% in some series [5]). Clip-guided BCS has proven to be safe with a local recurrence control rate of 98.6% at five years [6].

Likewise, conservative management of the axilla is possible in selected patients. According to NCCN guidelines [7], before NACT a clip or a tattoo should be placed in the FNAB-proven positive lymph nodes to allow targeted excision at the time of surgery. In fact, among node-positive patients becoming clinically node-negative after NACT, sentinel lymph node biopsy alone has shown a >10% false-negative rate. The removal of multiple lymph nodes, identified with a dual tracer along with the marked node, can reduce this rate to 2% [8]–6.8% [9]. Sentinel lymph node biopsy in patients who experienced axillary rCR after NACT avoided up to 47.6% of axillary dissections [10]. In case of non-rCR at preoperative imaging, or if metastases or isolated tumor cells (ITC) in any of the excised lymph nodes are identified after NACT, an axillary dissection must be performed.

However, general agreement on axillary management has not been reached yet and often depends on different local guidelines [11].

### 1.2. Different Marking Techniques

Multiple tumor localization techniques have been studied. The devices used for labeling lesions in clinical practice include tattoo ink, radioactive iodine seeds, ultrasound detectable clips, and magnetic implants.

Cutaneous tattoos are among the cheapest and most rapid techniques used to mark breast lesions, but regarding the axilla the identification of a normal-sized lymph node based on a superficial tattoo can be difficult.

Another marking method is the intralesional injection of a charcoal suspension. During surgery, the target area is visually identified by the dark stain left on the patients’ skin. Major drawbacks are the risk of color migration to other lymph nodes or to the perinodal fat and the risk of confusing the charcoal suspension with the blue dye used for sentinel lymph node identification [12,13].

Radioactive markers can be used as well. The MARI technique (“marking the axillary lymph node with radioactive iodine seeds”) [14] consists of the insertion of a iodine 125 radioactive seed into the affected lymph node before NACT. It is an effective technique, but it requires complex safety regulations.

Then, magnetic implants such as the MagSeed^®^ have been approved for long-term implantation in any soft tissue, thus allowing the direct insertion of the magnetic seed in the lesion before NACT. Reitsamer et al. [15], in their series of 40 patients, demonstrated a detection rate of previously marked lymph nodes of 100%. In this study, the positive lymph node was marked with a radiopaque clip before NACT and the clipped node was then targeted with the magnetic seed the day before the intervention and detected during surgery with a magnetic probe.

Last, radiopaque clips are a commonly used as marking technique in the NACT setting. There are different kinds of clips that can be generically divided into two categories [16]: metallic clips (made of steel, titanium, or alloys) and radiopaque clips made of metallic material covered with biocompatible hygroscopic material (collagen, polylactic or polyglycolic acid). The first group proved to have better ultrasound visibility compared to the second, but it is more likely to produce artifacts on MRI [17].

Since neoadjuvant chemotherapy lasts approximately 6 months, the radiological visibility of the tissue marker at the end of medical treatment remains a key aspect. At the time of surgery, radiopaque clips visibility is optimal, ranging between 83 and 100% according to different studies [18,19].

Clips are used to mark both the breast lesion and the positive lymph nodes biopsied before NACT. A feasibility study on twenty patients [20], has demonstrated a high identification rate (95.8%) of clip-marked lymph nodes at the time of surgery.

In a German study, based on a bigger sample, the target lymph node was successfully removed in 329 out of 423 patients, mostly after wire localization, resulting in an overall removal rate of 78% [21].

The targeted lesion can also be identified with a radiofrequency identification (RFID) technique. This method has been validated to identify non-palpable lesions eligible for upfront surgery and a recent study on 10 patients described the feasibility of this technique also to identify targeted lymph-node with a high success rate [22]. Only initial and limited data are available on the use of RFID in the neoadjuvant setting. Even if this method seems to be highly precise (the LOCalizer probe used to identify the chip displays the distance of the probe from the tag in real-time), it has high costs and can cause possible MRI artifacts. Moreover, in some patients with a pacemaker or other devices, the safety of this technique is not proven.

Once inserted, the marking clip can dislocate from the injection site [23], often more than 10 mm. For this reason, most of the studies that analyzed this aspect underline the importance of a post-insertion mammographic or sonographic control [5,24,25].

Regarding clipped lymph nodes, data are poorer, but some report a risk of dislocation of the clip in the perinodal fat tissue in about 30% of cases [26].

## 2. Materials and Methods

This study included all patients who underwent NACT and subsequent surgery from January 2018 to January 2022 at Mauriziano Umberto I Hospital in Turin. The selection of patients and their clinical management followed the recommendations of the guidelines in force. Data related to the feasibility of clip insertion, after-treatment visibility, localization, and successful removal during surgery were collected in a dedicated database. The primary outcome was evaluating the feasibility of this marking technique for both breast and axillary surgery. The secondary outcome was the role of this technique on surgical outcomes.

### 2.1. Selection of Patients

Patients eligible to NACT according to AIOM [27] and NCCN [7] guidelines were selected. All cases were discussed in a multidisciplinary board and had whole-body staging for distant metastases before NACT. In all cases, a breast MRI was performed to assess the initial tumor extension and another MRI was performed at the end of NACT to assess the final radiological response.

Inclusion criteria for the insertion of breast clips were unifocal disease and the feasibility of BCS in case of downstaging after NACT. Axillary clips were placed only in patients with cN1 node involvement at diagnosis.

Carcinomatous mastitis, multifocal disease, extensive in situ disease, and node involvement cN2-3 at diagnosis were exclusion criteria for the insertion of the clip in the breast lesion or the lymph node, respectively.

### 2.2. Choice of Tissue Marker

The insertion of a radiopaque clip was chosen among all tissue marking techniques. Three different kinds of clips were inserted (1 Mammostar, 1 Hydramark, and 52 Ultracor Twirl). UltraCor Twirl clip was used in the majority of cases (94.5%). This clip can be placed inside the lesion under ultrasound or stereotaxic guidance if lesions were not visible at the ultrasound. It is a ring-shaped wire made of nitinol, a metal alloy of titanium and nickel; the main contraindication to the use of this device is nickel allergy. Nitinol has many physical characteristics that make it suitable for medical uses, such as its shape memory, elasticity, and biocompatibility.

This marker is MRI compatible, provided that the static magnetic field is 1.5 or 3 tesla; the spatial gradient magnetic field is a maximum of 4000 gauss/cm (40 T/m) or less, and the maximum mediated specific absorption rate (SAR) on the internal body reported by the MR system is 4 w/kg.

Non-clinical tests have shown how the marker can cause imaging artifacts. These changes may extend to approximately 10 mm from the marker [28].

All tissue markers were placed by a specialized radiologist in the breast lesion or the axilla, targeting the biopsied lymph node with a positive result.

**Figure 1:**
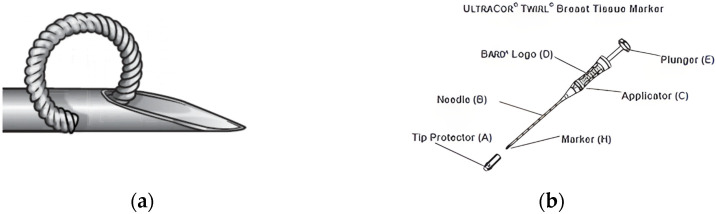
Ultracor Twirl clip (**a**) and applicator (**b**).

As shown in Figure 1, the disposable applicator features a 17 G × 10 cm blunt needle with centimeter depth indicators and a locking plunger. The radiopaque clip is released from the blunt needle tip without a skin incision. The procedure requires a few minutes and can be carried out after the injection of a local anesthetic at the skin entry site. As for all intramammary devices, the possible adverse effects include hematoma, hemorrhage, infection, allergic reaction, marker migration, and pain.

Although there is no established indication of where to place the clip inside the lesion, in agreement with the radiologist, all clips were intended to be placed in the center of the lesion. After the procedure, ultrasound images of the clip and its position inside the lesion/lymph node were acquired and described in the medical report.

### 2.3. Pre-Operative Evaluation

At the end of the NACT, the tumor response was evaluated through breast MRI. Surgery was scheduled approximately 2–4 weeks after the end of medical treatment (in case of use of weekly taxanes), according to recent guidelines [29].

If the MRI showed a radiological complete response and BCS was indicated, the clip was used as a landmark. A localizing metal wire was inserted, and the clip was removed during surgery along with a part of the surrounding parenchyma.

Surgery on the axilla was chosen based on the initial and final axillary status.

- No clinical axillary involvement at diagnosis: sentinel lymph node biopsy after NACT was performed.
- Clinical axillary involvement at diagnosis and still evident after NACT: axillary dissection was performed.
- Clinical axillary involvement at diagnosis with a radiological response after NACT: modified sentinel lymph nodes biopsy was performed (based on the retrieval of at least 3 lymph nodes, identified with a dual tracer technique, and the excision of the clipped lymph node—which was biopsied with positive results before NACT).

A definitive pathological assessment was performed and response to NACT was described following Pinder classification.

## 3. Results

### 3.1. Population

Fifty-three patients underwent NACT between January 2018 and January 2022 at Mauriziano Umberto I Hospital in Turin. Two patients were excluded from the analysis because surgery was performed in another institution. Data were completely recorded for 51 patients; among them, three patients had bilateral disease at diagnosis (total: 54 tumors).

The median age of patients who underwent NACT was 50.4 years. The majority of tumors (92.5%) had more than 2 cm extent (50/54), and 70.4% had homolateral lymph-node involvement at diagnosis (38/54). Immunohistochemical evaluation of receptor expression was performed on the biopsy specimens of 52 breast lesions. Twenty-five percent of lesions were categorized as triple-negative breast cancers, (TNBC) (13/52), 50% as HER2-positive tumors (26/52), and the remaining 25% as ER-positive/HER negative cancers (13/52), the majority of them having node involvement (10/13). All lesions were nonspecial type tumors.

In 13 patients no clip was placed because of advanced disease at diagnosis, multicentric disease, or >N1 nodal involvement, and no conservative surgery was achievable regardless of response to NACT. Therefore, they were not considered for the following analysis.

**Figure 2:**
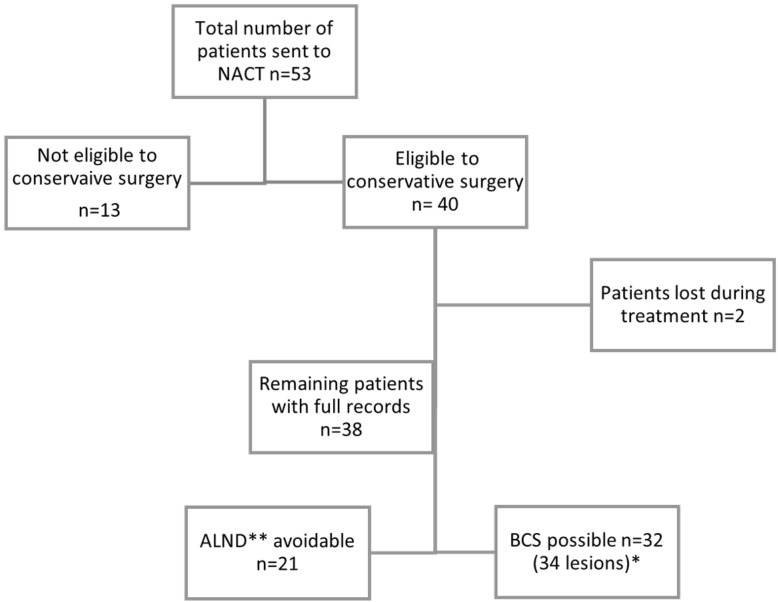
Study population. * Two patients had bilateral disease eligible to BSC, ** axillary lymph node dissection.

In 38 out of 51 patients a breast and/or axillary clip was placed for a total of 55 clips. In particular, 34 clips were placed in the breast lesion and 21 in the lymph node previously biopsied with a positive result (Figure 2). Two patients with bilateral disease at diagnosis underwent bilateral clip insertion. In 15 cases a clip was placed both in the breast lesion and in the homolateral adenopathy, in 19 cases the clip was placed only in the breast lesion and in 6 cases the clip was inserted only in the axillary lymph node (Table 1).

Among 55 clips, 1 Mammostar, 1 Hydramark, and 52 Ultracor Twirl were used. In one case, no information was provided about the brand of the clip.

To guarantee high procedural standards, all clips were inserted by one trained radiologist, proving the technique to be replicable in terms of duration (it takes about 20 min; range 15–25 min) and success.

**Figure 3:**
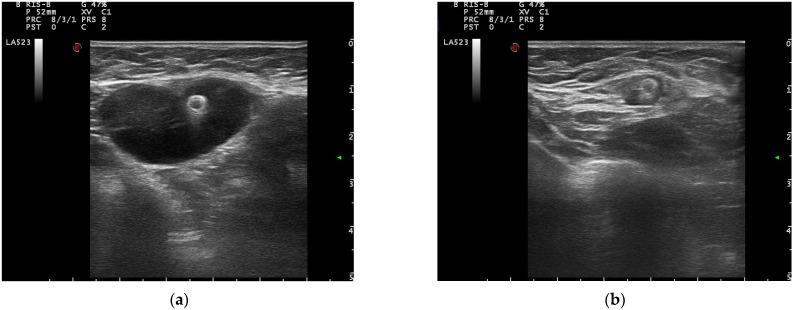
(**a**) Clip in breast lesion; (**b**) clip in a lymph node.

Once the clip is inserted, ultrasound images were acquired, and the localization of the clip and its position within the lesion were thoroughly described (Figure 3). In 87% of cases, the clip was correctly placed in the center of the lesion. In the other cases the clip was placed inside the lesion but in a peripherical position and the distance from the center of the lesion was accurately described. No severe adverse events were recorded.

The average duration of chemotherapy was 154 days and the average time from clip insertion to surgery was 178.5 days. During chemotherapy, no clip-related side effects were described.

### 3.2. Pre-Operative Clip Visibility, Localization, and Surgical Management

After chemotherapy, 53.7% of lesions achieved a breast rCR (29/54) and in 47.3% (18/38) of cases, an axillary rCR was observed.

Sixty-five percent (22/34) of the breast lesions in which the clip was placed and 71.4% (15/21) of clipped positive lymph nodes reached an rCR. In four cases, despite the presence of the clip and the rCR, mastectomy was performed based on the choice of the patients. Breast-conserving surgery was eventually performed in 18 cases.

In 35 cases preoperative localization of the clip, either in the breast or in the axilla, was required.

Thirty-two out of thirty-five clips were visible on ultrasound at the end of NACT (91%). Two clips were identified in mammograms whereas in one case the clip was not detected at all (but then found in the surgical specimen).

Wire localization was used to assure a correct removal of the clip during surgery with minimal discomfort for the patients and high effectiveness. In two cases the clips were superficial so that a skin tattoo was sufficient to identify them without wire insertion.

Dislocation of the clip was described in two cases, both involving the axillary clip. In these cases, the radiopaque marker was identified outside the lymph node at a distance of 30 mm and 20 mm.

After NACT, 26 BCS were performed. Sixty-nine percent of them (18/26) had an rCR: hence, conservative management instead of mastectomy was possible only because of the presence of the clip.

Intra-operatory pathological assessment required margins shaving in one case. In the other four cases, surgical enlargements were made on clinical suspicion. No enlargements were necessary after the definitive pathological assessment.

Regarding axillary management, an rCR on lymph nodes was obtained in 15 cases. A clinical decision was made to proceed directly to ALND in two patients. Thirteen patients underwent modified sentinel lymph node biopsy with the retrieval of multiple sentinel lymph nodes (SL) identified with dual tracer and excision of the previously clipped node. The average number of removed lymph nodes was 3.7.

In four cases metastasis or micrometastases were detected and ALND was performed. ALND was avoided in 42.8% (9/21) of initially cN1 patients, and in 69.2% of cases in which SNLB was attempted (9/13). In 46% of cases (6/13) the sentinel lymph nodes and the clipped lymph node (positive at diagnosis) did not correspond.

As collateral data, we observed a breast pathological complete response (pCR) rate, defined as ypT0/Tis, in 48.1% (26/54) of lesions and an axillary pCR (ypN0) in 44.7% (17/38) of initially node positive patients. Among them 34% (13/38) reached a pCR both in the breast and in the axilla. The majority of lesions that reached a breast and/or axillary pCR showed HER2 overexpression or were TNBC. We observed a 73% (19/26) of breast/axillary pCR in the first group, and a 61.5% (8/13) among TNBC. Only one pCR was shown among ER positive/HER2 negative cancers (7%).

## 4. Discussion

### 4.1. Selection of Patients

Patients eligible for NACT were selected according to the national and international guidelines in force, as 75% of lesions were triple-negative or HER2 positive cancers. Between January 2018 and January 2022, 53 patients received NACT. Within our patient cohort, we have observed a progressive increase in the number of patients undergoing NACT in recent years due to better patient selection in our clinical practice. Even if randomized controlled trials showed similar long-term outcomes between patients who received the same chemotherapy scheme before or after surgery [30], better survival outcomes have been demonstrated in high-risk patients with TNBC or HER2 positive diseases who achieved a pathological complete response (pCR) after NACT. Moreover, the evaluation of the response to NACT allows to tailor the subsequent treatments when a pCR is not achieved, thus improving the prognosis of this group of patients [31].

As reported by von Minckwitz et al. [32] in a review of seven trials including more than 6000 patients, disease-free survival was significantly superior in those patients with no invasive and no in situ residuals. According to the CTNeoBC analysis [3] this association was strongest in patients with TN and HER2-positive breast cancer.

In our study population, around 25% of lesions were luminal B tumors HER2 negative. The choice between either pre-operative or post-operative systemic treatment in ER positive-HER2 negative breast cancer is more difficult, as there is no direct evidence of benefit of NACT in this group [33], nor is available a targeted post-neoadjuvant treatment in those patients who do not achieve pCR. To date, NACT represents the only chance for cN1 patients to avoid axillary dissection.

### 4.2. Clip Positioning and Visibility

The use of radiopaque clips proved to be safe, the positioning procedure was easy, and no severe adverse event was recorded. Moreover, the insertion of the clip required the patient to go to the hospital only one extra time, thus not being too burdensome.

The ultrasound visibility of the clip at the end of chemotherapy appeared to be optimal, being possible in nearly 100% of cases. In only three cases the clip inserted was not detected by ultrasound and required mammographic evaluation. The clip was therefore visible after an average time from insertion of 5.9 months and up to a maximum of 7.5 months. These results are partially confirmed in literature as the ultrasound visibility varies between the type of clips, which differ in shape and size. Pinkey et al. [34] analyzed the ultrasound visibility of five different clips at 6 and 12 weeks after placement on a sample of 23 patients. They found significant differences among different devices (SenoMark, Gel Mark, HydroMARK, SecurMark, and UltraClip Enhanced Coil). Another study [19] compared the visibility of other clips at the end of NACT: the UltraClip marker was found to be less visible than Ligaclip e Comark, which had ultrasound visibility of 91.1% and 86.9%, respectively.

The chosen marker (UltraCor Twirl clip) showed high ultrasound visibility on animal tissue models [35] as well as in clinical settings. A recent study from Lim et al. [36] further confirms the good ultrasound visibility of this device. Out of 25 clips inserted in a population of 14 patients, the 13 UltraCor Twirls proved to be better than other devices, with a 100% ultrasound visibility.

In our study, we observed an excellent rate of preoperative wire localization of the marking clip (both on breast and axillary clips), with only a single failure that made it necessary to insert a second wire. Contrariwise, other studies, which used the same technique, have reported a variable number of failures, especially for the localization of axillary clips, with identification rates in the operating setting ranging from 70% to 97% [37]. Hartmann et al. [26], on a series of 30 patients, had an identification rate of clipped lymph node of 70.8%. Possible suggested explanations were the accidental dislocation of the reference wire due to the patient’s movements or a primitive dislocation of the clip in the fat surrounding the target lymph node.

Only two clip dislocation events were found in our series. In one of them, the clip initially placed inside the lymph node was then found next to it. To solve this problem, two reference wires were positioned (one localizing the clip and the other in correspondence with the lymph node). Both the clip and the lymph node were removed during surgery.

To date, there are few studies analyzing the rate of clip dislocation at the end of NACT, whereas some studies, as reported by Pinkney et al. [23], describe rates of early dislocation (in post-positioning control) that can affect up to 40% of clips inserted.

In the neoadjuvant setting, our experience corroborates the findings of Schulz-Wendtland et al. [25], demonstrating no evidence of significant clip migration after completion of neoadjuvant chemotherapy.

### 4.3. Pre-Operative Evaluation and Surgical Management

Pre-operative MRI showed a breast rCR rate of 53.7% and an axillary rCR rate of 47.3%. Similar findings were presented by Weber et al. [38]. The authors conducted a study on 129 patients enrolled between June 2014 and August 2015 and observed a breast radiological response rate of 31.8%, while the lymph node response rose to 51% among initially node-positive patients.

One of the aims of NACT is to allow conservative surgery. A study-level meta-analysis by Houssami et al. [39] on 30 primary studies and 11,695 patients demonstrated a higher percentage of BCS (58.1%) compared to mastectomy (39.4%) in patients who underwent NACT. A more recent metanalysis on 10 trials involving 4756 patients showed that patients treated with NACT had an increased frequency of breast-conserving surgery (65%) compared to patients treated with adjuvant chemotherapy (49%) [40].

In our findings, a total of 26 BCS were performed. In at least 18 of them, a mastectomy would have been necessary based only on pre-NACT evaluation. In 69.2% of cases, BCS was made possible only thanks to the insertion of the clip before the NACT, since the radiological post-treatment evaluation did not provide any indication of the site of the primitive lesion. In these cases, in the absence of a tissue marker, a mastectomy would have been necessary.

Among node-positive patients at diagnosis who were eligible for conservative surgery in case of clinical downstaging of the axilla, 42.8% avoided axillary dissection. Similar findings are described in the trials conducted by Mamtani et al. [10] and Galimberti et al. [41] who avoided axillary dissection in 48% and 47.6% of cases, respectively.

Another important aspect is the correspondence between the sentinel lymph nodes and the clipped node. In our series, the clipped lymph nodes differed from the sentinel ones in up to 46% of cases. Other studies had a variable percentage of non-correspondence, varying from 23% [8] to up to 40% [42]. Marking the positive node at diagnosis is therefore fundamental to guarantee an adequate analysis of axillary status after NACT.

Pathological complete response, defined on final pathological assessment, was comparable to other studies conducted on bigger samples that showed variables pCR rates on the breast of 27–37% and on the axilla of 38–49%. As expected, pCR rates were higher in lesions with HER2 overexpression and triple-negative breast cancers [10,43].

## 5. Limitations and Strengths

The limitations of the study include the low number of patients and the lack of a control group. In fact, our study included only 53 patients receiving neoadjuvant chemotherapy over 4 years, and the absence of a control group, although not affecting the primary objective of this study, did not allow a comparison between retrieval techniques. Furthermore, it was not possible to conduct a cost-benefit analysis of the technique.

However, we can point out that similar studies on the feasibility of marking techniques, clip visibility and localization methods, have a comparable or lower number of patients involved. Moreover, we observed an increasing number of patients referred to NACT over the recent years primarily due to the practice shift induced by the consensus conference of St. Gallen in March 2019, thus expecting a further growth in our study population in the future.

## 6. Conclusions

To date, surgery after NACT is mandatory, but its extension can be modulated based on pre- and post-NACT evaluations. In our experience, the use of radiopaque clips as a marker of the breast lesion or the positive nodes at diagnosis has proven to be a safe and effective method to guide surgical management in case of a radiological complete response. Radiopaque clips showed optimal characteristics in terms of practical features. Moreover, they allowed to increase the number of breast-conserving surgery and to reduce the number of axillary dissections and related morbidity. In our findings, tissue markers were also fundamental to guarantee the identification of initially positive nodes which did not always correspond to the sentinel nodes excised.

## Figures and Tables

**Table 1:** Number and localization of clips.

Breast + axillary clips	30 (54.5%)
Only in breast	19 (34.5%)
Only in lymph node	6 (11%)
Clips placed in breast lesions	34 (61.8%)
Clips placed in lymph nodes	21 (38.2%)
Total number of clips	55 (100%)

## Data Availability

The data presented in this study are available on request from the corresponding author on reasonable request. The data are not publicly available due to privacy policies.
